# A Qualitative Study of Senior Residents’ Strategies to Prepare for Unsupervised Practice

**DOI:** 10.5811/westjem.48914

**Published:** 2025-11-26

**Authors:** Max Griffith, Alexander Garrett, Bjorn K. Watsjold, Joshua Jauregui, Mallory Davis, Jonathan S. Ilgen

**Affiliations:** * University of Washington, Department of Emergency Medicine, Seattle, Washington; † University of Michigan, Ann Arbor, Department of Michigan, Ann Arbor, Michigan

## Abstract

**Introduction:**

As emergency medicine (EM) residents prepare for the transition into unsupervised practice, their focus shifts from demonstrating competencies within familiar training environments to anticipating their new roles and responsibilities as attending physicians, often in unfamiliar settings. Using the self-regulated learning framework, we explored how senior EM residents proactively identify goals and enact learning strategies leading up to the transition from residency into unsupervised practice.

**Methods:**

In this study we used a constructivist grounded theory approach, interviewing EM residents in their final year of training at two residency programs. Using the self-regulated learning framework as a sensitizing concept for analysis, we conducted inductive, line-by-line coding of interview transcripts and grouped codes into categories. Theoretical sufficiency was reached after 12 interviews, with four subsequent interviews producing no divergent or disconfirming examples.

**Results:**

We interviewed16 senior residents about their self-regulated learning approaches to preparing for unsupervised practice. Participants identified two types of gaps that they sought to address prior to entering practice: knowledge/skill gaps, and autonomy gaps. We employed specific workplace learning strategies to address each type of gap, which we have termed cherry-picking, case-based hypotheticals, parachuting, and making the call, and reflection on both internal and external sources of feedback to assess the effectiveness of these learning strategies. This study presents participants’ identification of gaps in their residency training, their learning strategies, and reflections as cyclical processes of self-regulated learning.

**Conclusion:**

In their final months of training EM residents strategically leverage learning strategies to bridge gaps between their self-assessed capabilities and those they anticipate needing to succeed in unsupervised practice. These findings show that trainees have agency in how they use goal setting, strategic actions, and ongoing reflection to prepare themselves for unsupervised practice. Our findings also suggest tailored approaches whereby programs can support learning experiences that foster senior residents’ agency when preparing for the challenges of future practice.

## INTRODUCTION

Competency-based medical education frameworks provide scaffolding and accountability to ensure that emergency medicine (EM) trainees develop the necessary knowledge and skills for unsupervised practice.^1^,^2^ While competency-based medical education frameworks provide a roadmap for residents to deliberately practice the core elements of EM, graduates of EM training programs often lament the inevitability of encountering new challenges when entering practice.^3^,^4^ This suggests that the training experiences that advance residents’ competencies (what a resident does to demonstrate their abilities) must be done in conjunction with efforts to advance residents’ capabilities (the things they can think or do in future practice.)^5^ While competencies are often embedded in the tools that training programs use to assess residents,^1^,^6^,^7^ capability development requires trainees to engage in dynamic self-assessment^8^ to consider what they can work on now to prepare themselves for future transitions. A capability approach looks beyond training residents who are simply competent, aiming instead to develop trainees who can self-diagnose their future learning needs and enact learning strategies to achieve their goals.^9^–^11^

Self-regulated learning (SRL) provides a framework to study how senior residents approach workplace learning to prepare for their transitions into unsupervised practice.^12^ The SRL theory proposes that individuals are “metacognitively, motivationally, and behaviorally active participants in their own learning process.”^13^ This provides a structure to consider how residents might assess their abilities and modulate their activities during training.^14^ These SRL behaviors are often depicted as a cycle whereby individuals set goals, employ learning strategies to attain these goals, and reflect on their progress.^14^ This cycle is context-dependent, shaped by learner characteristics (eg, knowledge, prior experiences, emotions, and confidence) as well as by the learning environment (structure, supports, and cultural expectations).^15^ Learner-related factors such as autonomy, efficacy, and accumulated experience have been shown to support engagement with SRL,^16^ suggesting that residents in the final months of training have nuanced and mature self-regulated learning habits.

The end of residency training is a compelling period to examine SRL as it relates to capability development. As residents prepare for the transition into unsupervised practice, their focus shifts from demonstrating advanced competencies within familiar training environments^7^ to anticipating their performance with new roles and responsibilities as attending physicians, often in unfamiliar practice environments.^17^–^21^ In recent work exploring how senior EM residents conceptualized their preparedness for unsupervised practice, we found that trainees were cognizant of the inevitable mismatch between what they learned in training and what they would be expected to do in unsupervised practice.^3^

We were struck by trainees’ sense of agency in their reflections,^22^ particularly by how they set goals and leveraged their learning environments to create learning strategies that addressed their anticipated future practice needs. Recognizing that these findings have not been described previously in the literature, we returned to our data using the lens of SRL to explore how senior residents proactively identified goals and enacted learning strategies specific to their transitions from residency into unsupervised practice. By elaborating these strategies, we hope to provide insights that educators can use to tailor their support for senior trainees during these important transition periods.

**Population Health Research Capsule**

What do we already know about this issue?

*Emergency medicine (EM) residency graduates are often anxious about the unfamiliar clinical problems that they will encounter in unsupervised practice.*

What was the research question?

*What workplace learning strategies do EM senior residents use to prepare themselves for unsupervised practice?*

What was the major finding of the study?

*We describe self-regulated learning strategies: cherry picking, case-based hypotheticals, parachuting, and making the call.*

How does this improve population health?

*These learning strategies can improve new physicians’ preparedness to treat patients without supervision in a variety of clinical settings.*

## METHODS

We chose a constructivist grounded theory approach for this qualitative study, a methodology appropriate to study a complex social process about which relatively little is already known.^23^ We assembled a research team with a range of expertise and experiences, recognizing the importance of subjectivity in our processes of building theory through analyses of our participants’ narratives.^24^ The author group consisted of emergency clinician educators from both participating institutions, all of whom regularly supervise senior residents. We approached this study with an understanding of the challenges and affordances of the emergency department (ED) learning environment. At the time of data collection, three of the authors (MG, AG, MD) were each one year removed from residency, which allowed them to reflect on their recent training experiences as well as the challenges of working as new attending physicians.

### Conceptual framework

This study is part of a larger program of research about how senior residents prepare themselves for unsupervised practice. In prior work,^3^ we described how senior residents adopted a future-oriented, capability approach to workplace learning,^5^ using past training experiences as starting points to engage with unforeseen problems in practice. Our participants recognized that uncertainty and unfamiliar problems were inevitabilities of future practice, and defined preparedness in terms of the skills and approaches that would enable them to capably adapt to unforeseen challenges. This understanding led us to consider how senior residents might proactively use their final months of training to further these goals of adaptability^25^,^26^ and capability development.^5^ For this study, we used the SRL framework as a sensitizing concept for additional analysis, focusing on how participants described their dynamic processes of setting goals, strategizing for workplace learning, and monitoring their progress.^13^,^14^,^27^,^28^

### Setting, Population and Sampling Strategy

We recruited EM residents in their final year of training at two four-year residency programs, each housed within large, academic healthcare centers with Level I trauma designation and rotations between multiple clinical sites. Fourth-year trainees in each program work a combination of “pre-attending” shifts, in which they supervise junior trainees with an attending physician also present, and primary shifts, during which they manage patients directly with attending supervision. We chose this cohort because of their proximity to their transition into unsupervised practice as well as their familiarity with their residency learning environments accumulated over three prior years of training. We sampled from geographically distinct areas (Midwest and Western United States) to account for local practice patterns and workplace cultures.

Interviews occurred between April–June 2023, when participants were still immersed in training but had solidified their immediate post-residency career plans. Email invitations were sent to all 28 eligible residents, with assurances that data would be deidentified before analysis and that participation would have no bearing on their standing within their programs. Interviews were scheduled in the order that residents responded. Participants were reimbursed with a $100 gift card. This study was reviewed and deemed exempt by institutional review boards at both sites.

### Procedures

The principal investigator (MG) used videoconferencing software (Zoom Video Communications, Inc., San Jose, CA) to conduct individual virtual interviews. We piloted the interview guide (Supplemental material) with two senior trainees not participating in this study, and modified questions for clarity. We then conducted semi-structured interviews, asking participants about their career plans after residency, what it means to be “prepared” for unsupervised practice, and what challenges they anticipated as they entered work in new contexts. We probed about how residents developed learning goals and enacted specific workplace learning strategies to prepare themselves for the experiences they anticipated in unsupervised practice. We used a professional transcription service (Rev.com. Inc., Austin, TX) to transcribe recordings, which MG then deidentified and checked for accuracy prior to analysis by the group.

### Analysis

The entire research team coded four initial interview transcripts line-by-line to inductively develop a preliminary codebook, after which we agreed on a focused set of codes for the remaining data. Two investigators (MG and AG) then coded all transcripts with Dedoose (Social Cultural Research Consultants, LLC, Los Angeles, CA), using memos to keep track of conflicting examples or ideas requiring more exploration, and meeting frequently to discuss coding discrepancies. The entire group met periodically to resolve differing interpretations of the data, discuss relationships between codes, and group codes into categories. Drawing from these categories, we constructed theory as defined by Charmaz: to “present arguments about the world and the relationships within it.” 28,29^(p128)^ Our coding framework sufficiently captured our construct of interest after 12 interviews. Finding no divergent or disconfirming examples in four subsequent interviews, we deemed our dataset sufficient for the study’s aims.^30^

## RESULTS

We interviewed 16 EM senior residents (ten and six from each respective residency program; nine women and seven men). These residents had accepted positions to work clinically at a variety of community, academic, county, and community-academic hybrid sites, often splitting time between multiple clinical practice settings. Three participants were entering EM fellowships but with clinical roles as attending physicians. One participant was slated to start work as a critical care fellow, albeit with opportunities to work unsupervised shifts in the hospital’s ED. Across these interviews, participants shared a view that the resources, practice patterns, and pathologies characteristic of their training sites did not reflect clinical practice in most other settings. This perception shaped their learning goals for the final months of training, motivating them to develop learning strategies that bridged gaps between the capabilities they had developed in their existing training contexts and the skills they anticipated needing in unsupervised practice.

We identified learning strategies in our initial coding of participants’ stories. We then applied SRL as a theoretical lens, which allowed us to arrange those strategies into cycles involving an initial planning phase, in which participants self-identified gaps, an action phase where they deployed learning strategies to address these gaps, and a phase of reflection on their progress. Finally, we divided these cycles as we recognized that they addressed two types of gaps—knowledge/skill gaps and autonomy gaps—to represent how our participants engaged in SRL cycles as a response to their impending clinical transitions.

### Gaps in Participants’ Knowledge and Skills

In reflecting on their readiness to enter unsupervised practice, participants identified crucial gaps in their abilities to understand and manage unfamiliar clinical problems. These perceived gaps stemmed from limitations to the pathologies and patient complaints that they were exposed to during training, due to the time-limited nature of training as well as the affordances and limitations of working at an academic healthcare center. Many participants anticipated managing conditions with less input from specialists than they did at their current academic healthcare centers and worried they might be insufficiently prepared to manage these problems on their own. As Participant 11 reflected, “we just have so many consultants …we take a backseat on a lot of things.” Participants also worried that structures within their training environments—such as the tendency for nurse practitioners or physician assistants to care for patients with low-acuity complaints—buffered them from the realities of community practice:

I feel like we often are protected from the urgent care complaints… just because we have great mid-levels, and also it just doesn’t feel like that type of community medicine comes in that much. (Participant 6)

#### Learning Strategies: Cherry-picking and Case-based Hypotheticals

Participants adopted two strategies to address perceived knowledge and skill gaps in their training. For each, they strategically leveraged the resources of their training environments to build confidence that they could handle the anticipated challenges of their future practice. First, participants described acts of cherry- picking, selectively engaging with clinical tasks that addressed gaps in their knowledge and skills. They seemed to view their last months of training as an opportunity to seek out pathologies and procedures in areas where they felt inadequately prepared, at times prioritizing these over tasks that they viewed as less educationally enriching. For example, after self-identifying a need for more experience with orthopedic injuries, Participant 15 described an instance in which they intentionally sought out orthopedic experiences on shift.

“I knew there was a bunch of ortho stuff going into the department, and so I just said to [my attending] … ‘you won’t see me for a while. I’m going to spend the next few hours doing ortho stuff.’ And, so, I just went along and properly learned some better techniques.”

Yet residents often discussed the difficult balance between cherry-picking and expectations of patient throughput. Several participants noted that they had not felt empowered to focus on gaps in their learning until the final years of their training. They attributed this greater sense of agency to their familiarity with the clinical workflow, their comfort with their clinical supervisors, and the sense of urgency imparted by the upcoming transition to unsupervised practice. As Participant 6 explained:

I was able to recognize that, after I don’t know how many years … if the patient needs to be seen because they’re super sick, then happily I’ll see them and I’ll see them fast. But you know, the eighth abdominal pain can sit for 20 minutes while I focus on the critical care or the pathology that I don’t really understand or recognize yet, because that’s more important.

A second strategy that participants used to address their gaps in knowledge and skills was case-based hypotheticals, moments when they deliberately slowed down and stretched a clinical experience to consider alternative approaches or dimensions they might face. Participant 7 used the expression “mental war-gaming” to describe their process of thinking through a range of case-specific “if this happens, what would I do?” hypotheticals with the help of their supervisor. Another participant elaborated on how considering hypotheticals with trusted supervisors helped them to feel more confident tackling novel problems in unsupervised practice:

Just trying to go through every line of how this [case] could turn out, so that when it does turn out that way [next year], I have a good frame of reference of what the attending would do … I think that just doubles your number. You’re essentially creating a new patient in your mind, right? … It’s not the unknown anymore.” (Participant 6)

#### Reflection: Programmatic Feedback

Participants often struggled with the poor alignment of external formative feedback sources—such as procedure logs, competency metrics, standardized tests, and evaluations from attendings—with their self-assessed knowledge and skills gaps. Despite this, they relied on programmatic assessment for feedback on these gaps and used it to calibrate their self-assessments. Participant 8 described the trust they placed in their program’s assessment processes:

I think you just have to really rely on the system that’s in place, and you have to trust the program leadership and the attendings to call you out when they think you are not ready in something.

Although these were recognized to be imperfect sources of feedback, many felt that nationally recognized milestone assessments and standardized tests were the best available substrate to reflect on their abilities to apply knowledge and skills in unsupervised practice.

I think to be a practicing emergency physician...you have to be able to pass the boards… Do you have enough information in your head that you’re not going to miss something glaringly obvious because you don’t know it? (Participant 14)

### Gaps in Participants’ Autonomy

As part of their transition to the attending physician role, participants anticipated a major leap in autonomy, with expectations of practicing independently and bearing the ultimate responsibility for decisions. Working without supervisory guidance was an anticipated source of stress and anxiety, as Participant 13 reflected:

I am going to be the adult in the room making all of these decisions. I don’t necessarily have the attending to say, ‘I’m not sure, let me go ask them.’ …Every call ... I’ll be the final one making it.

Confidence was frequently mentioned as an attribute needed for unsupervised practice, on par with any knowledge or skill set. Thus, participants strategized ways that they could use their workplace learning to build confidence in the decision-making they would need when working without supervision.

#### Learning Strategies: Parachuting and Making the Call

Participants adopted two learning strategies to engender confidence that they could engage in new tasks without the input of supervisors. They pushed themselves to expand their comfort zones in two ways: by trying new management approaches with supervisory support; or by deliberately seeking experiences where supervisory support felt absent. First, participants described acts of parachuting, deliberately seeking to safely try new things while still having the backstop provided by their clinical supervisors. They sought opportunities to trial unfamiliar approaches to patient care, for example treating with a medication they had little experience with or attempting a procedural method that they had not tried before, viewing these instances as moments when they could “widen [their] experience before getting too set in one way” (Participant 7). Participants were able to test their limits or attempt new things with reassurances that supervisors were available to help. Participant 5 described this support structure in the following way:

[T]his parachute that you know is there…no matter how much flexibility and how much autonomy our attendings give us, it’s very clear that there’s somebody to catch you if you fall.

Second, participants described deliberate efforts to adopt a mindset of working without supervisory support, pushing themselves to engage with high-stakes decisions before their supervisors provided input. Participant 13 described stroke evaluations as moments when they found opportunities to “make the call” with no supervisory guidance:

I try to jump on [stroke evaluations], whether or not the attending is there yet, and kind of make a call before that support comes in.

Making the call in this manner fostered self-reliance, and participants expected that experiences like this would lessen the stress of similar decisions in future unsupervised practice:

Whenever I come up with a patient and I’m like, ‘Oh, I need to ask about what to do.’ Then I just pause. I’m like, ‘No, I’m not going to ask. I’m going to figure out what I’m going to do and then present it that way.’ (Participant 2)

In addition to finding opportunities to practice their independent thinking during supervised shifts, several participants sought out authentic experiences of autonomy through moonlighting (working physician shifts for pay outside of their regular training, often unsupervised) to build confidence at the end of training.

#### Reflection: Internal Emotions and External Standards

Gauging whether they were ready for increased autonomy was difficult for participants, and they struggled to link their readiness to existing performance metrics within their residency training structures. Participants instead reflected on their emotional reactions in the workplace, as well as implicit feedback from their clinical supervisors as more holistic measures of whether they could be confidently autonomous. During high-stakes scenarios, participants took stock of their own internal emotional states, using these reactions as a measuring stick for whether they could handle the pressures of unsupervised practice. Participant 3 reflected on their comfort level while leading a pediatric code as evidence that they were ready to handle the increased autonomy:

Yeah, you know I felt uncomfortable in regard to it being a 3-year-old and it being stressful, but I didn’t feel out of my depth by any means. I felt like if that showed up at [a community hospital], even without a ton of surgeons, I felt like I would have been able to handle it.

Residents also cited their supervisors as external reference points that helped them reflect on their abilities to work autonomously. They described a practice of comparing their management plans to those of their supervisors, using instances of alignment or misalignment as ongoing sources of feedback. Working alongside attending physicians provided opportunities to identify a range of acceptable practice and evaluate decision-making against a trusted standard, either reaffirming or calling into question their sense of practice readiness.

“I am just quizzing myself against the attendings… you’re ready for more independent practice when all of your care decisions seem to fall within the range of people that you work with.” (Participant 9)

## DISCUSSION

Senior residents in this study described a range of strategies that they used to prepare themselves for unsupervised practice. Using SRL as a framework to interrogate these strategies, we showed how residents identified specific knowledge and skill gaps and the need to build confidence in their ability to work autonomously. They then strategically leveraged their familiarity with their training environments toward. tailored approaches that addressed these self-identified areas of development. To reflect on whether their learning strategies were effective, they looked to programmatic feedback, their own emotions, or their performance relative to their supervisors, using these sources of feedback to prepare themselves for new cycles of learning. Taken together, these acts of gap identification, strategic action, and reflection provided unique cycles of SRL specific to their upcoming transitions into practice (Figure).

This study adds to previous research on transitions into practice, which has historically focused on perspectives of attending physicians who have already entered their new professional roles.^18^,^31^–^35^ Teunissen and Westerman have argued that “a transition is not a moment, but rather a dynamic process,”^36^^(p45)^ encompassing the periods both leading up to and succeeding an advance in training. Other authors have questioned the very notion of “preparedness” for medical trainees, for whom performance depends heavily on the shifting contexts of their work environment.^37^ Our study provides a different perspective, namely that trainees can exert agency in how they use goal setting, strategic actions, and ongoing reflection to prepare themselves for the needs they anticipate in unsupervised practice, even if the specifics of their future practice remain unpredictable.

Our participants’ learning strategies align with recent work that has described SRL as context dependent.^12^,^15^ Senior trainees are more likely to employ nuanced learning strategies because they have developed competence with routine aspects of care over time within the contexts of their training environments.^5^ Furthermore, because they were familiar with their learning environments and supervisors, and perhaps because they felt a sense of urgency from the upcoming transition to practice, our participants seemed empowered to prioritize learning over service to the department. While many participants did reference a tension between “moving the meat” and taking time to grapple with new learning,^38^ senior residents in this study seemed more comfortable deferring non-emergent patient care to focus on high-yield learning opportunities. Our results also resonate with other models of self-regulated learning that have been studied in medical training, such as the master adaptive learner framework.^39^,^40^

Regan et al noted that master adaptive learners identify knowledge and skill gaps based on a combination of performance- and community-related cues; they triage learning opportunities based on complex considerations of needs, desires, and obligations; and they self-assess the effectiveness of their learning efforts.^41^ These authors also note the influence of context on SRL, with transitions in training prompting trainees to re-evaluate and adapt their learning strategies, and the specifics of each learning environment helping to shape the goals that trainees set for themselves.^42^ Our study’s participants showed similar processes of gap identification, learning, and self-assessment, all heavily influenced by the context of preparing for unsupervised practice during the final months of residency training. It would be informative to study how trainees develop specific learning goals regarding other significant transitions or milestones in training.

While these senior residents’ learning strategies were geared toward proactively seeking educational opportunities and fostering autonomy, supervisors clearly played a fundamental role in these experiences. Participants drew from supervisors’ support in both explicit and tacit ways—borrowing from supervisors’ experience to stretch their learning through hypotheticals that they might see in practice or engaging in new tasks equipped with their metaphorical parachutes. This framing expands the traditional framing of learner-centric SRL cycles toward paradigms such as “co-regulated learning” that emphasize the critical aspects of supervisory support at each step.^43^ Supervising physicians can guide senior residents’ goals for their workplace learning by highlighting differences between their training environments and their future practice settings, or spotlighting the skills that will maximize their confidence and future success. Supervisors can also guide senior residents to authentic experiences of autonomy and productive struggle,^44^ allowing them to grapple with clinical uncertainty while still being available for support.^45^

These findings present several important considerations for residency training programs. First, programs can more meaningfully consider residents’ individualized post-training needs, probing for perceived gaps and letting residents select (or even design) experiences that are likely to set them up for success in their unique practice contexts. Second, they can provide level-appropriate supervisory opportunities for senior residents while still in training, whether junior-attending shifts in the ED^46^ or moonlighting opportunities that allow them to assume the duties of an attending physician.^47^ Experiences when trainees are pushed to “make the call” clearly build confidence for future autonomy.

Finally, while residents in this study identified individualized learning goals, their means for reflection often involved less specific tools such as exam scores and procedure logs. This suggests opportunities for programs to better support each resident’s self-regulated learning efforts by helping them identify sources of feedback that meaningfully address their unique and contextualized learning goals. Our results suggest that residents gauge their own performance through multiple sources of feedback that are both explicit (eg, post-shift discussions with attendings, workplace-based assessments, and semi-annual reviews), and implicit (eg, social cues generated from their interactions with attendings, staff, and patients).^48^

## LIMITATIONS

Our results and analyses reflect several methodological decisions. We interviewed residents from two residency programs to allow for more diverse perspectives; however, both programs featured large Level I trauma centers and academic hospitals, and most of these residents were preparing for transitions to community practice. Thus, these participants’ actual and perceived knowledge gaps and learning goals may not reflect those of trainees from other programs. We focused only on residents’ strategies in anticipation of unsupervised practice; thus, our study was not designed to follow-up with participants after graduation to see whether these strategies were actually helpful in fostering preparedness.

It is important to note that this was a preplanned return to a dataset that was initially collected as part of a broader study about residents’ preparedness for practice.^3^ Returning to these data with a SRL lens enabled us to focus on specific dimensions of participants’ stories that were germane to cycles of learning, although this choice may have necessarily excluded other important aspects of their experiences.^49^ Finally, MG had professional relationships with the participants that he interviewed, either as a supervisor or former co-resident, and this may have shaped their responses as well as his interpretation of the data.

## CONCLUSION

Emergency medicine residents strategically leverage learning strategies in their final months of training to bridge perceived gaps between their self-assessed capabilities and those they anticipate needing to succeed in unsupervised practice. We present these strategies—cherry-picking, case-based hypotheticals, parachuting, and making the call—within cyclical processes of self-regulated learning, although they are notably codependent on supervisory support. These findings suggest tailored approaches whereby programs can support learning experiences that foster senior residents’ agency when preparing for the challenges of future practice.

## Supplementary Information



## Figures and Tables

**Figure:**
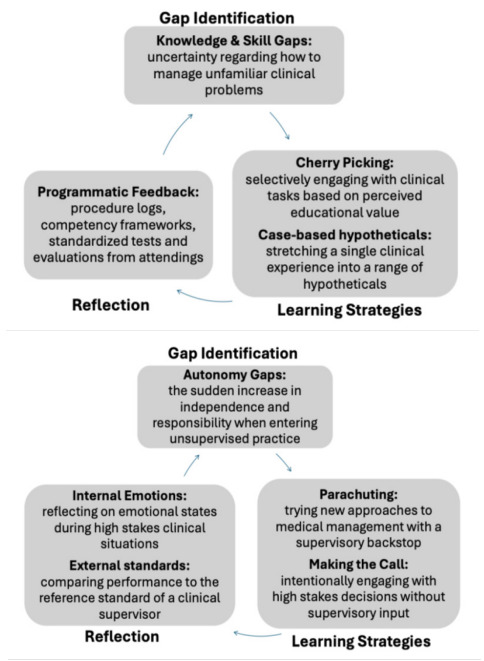
Gap identification, learning strategies, and means of reflection described by participants in a study of how senior residents in emergency medicine programs prepare for independent practice.

## References

[1] Hamstra SJ, Yamazaki K (2021). A validity framework for effective analysis and interpretation of milestones data. J Grad Med Educ.

[2] Frank JR, Snell L, Sherbino J (2015). CanMEDS 2015 Physician Competency Framework.

[3] Griffith M, Garrett A, Watsjold BK (2025). Ready, or not? A qualitative study of emergency medicine senior residents’ perspectives on preparing for practice. AEM Educ Train.

[4] Gamborg ML, Mylopoulos M, Mehlsen M (2024). Exploring adaptive expertise in residency: the (missed) opportunity of uncertainty. Adv Health Sci Educ Theory Pract.

[5] Teunissen PW, Watling CJ, Schrewe B (2021). Contextual competence: how residents develop competent performance in new settings. Med Educ.

[6] Cooney RR, Murano T, Ring H (2021). The Emergency Medicine Milestones 2.0: setting the stage for 2025 and beyond. AEM Educ Train.

[7] Holmboe ES, Yamazaki K, Nasca TJ (2020). Using longitudinal milestones data and learning analytics to facilitate the professional development of residents: early lessons from three specialties. Acad Med.

[8] Eva KW, Regehr G (2008). “I’ll never play professional football” and other fallacies of self-assessment. J Contin Educ Health Prof.

[9] Jain V, Oweis E, Woods CJ (2023). Mapping the distance: from competence to capability. ATS Sch.

[10] Stephenson J, Weil SW (1992). Quality in Learning: A Capability Approach in Higher Education.

[11] Mylopoulos M, Brydges R, Woods NN (2016). Preparation for future learning: a missing competency in health professions education?. Med Educ.

[12] van Houten-Schat MA, Berkhout JJ, van Dijk N (2018). Self-regulated learning in the clinical context: a systematic review. Med Educ.

[13] Zimmerman BJ (1986). Becoming a self-regulated learner: Which are the key subprocesses?. Contemp Educ Psychol.

[14] White CB, Gruppen LD, Fantone JC, Swanwick T (2013). Self-regulated learning in medical education. Understanding Medical Education.

[15] Brydges R, Butler D (2012). A reflective analysis of medical education research on self-regulation in learning and practice. Med Educ.

[16] Berkhout JJ, Helmich E, Teunissen PW (2015). Exploring the factors influencing clinical students’ self-regulated learning. Med Educ.

[17] Roten C, Baumgartner C, Mosimann S (2022). Challenges in the transition from resident to attending physician in general internal medicine: a multicenter qualitative study. BMC Med Educ.

[18] Collini A, Alstead E, Knight A (2023). “You may think that the consultants are great, and they know everything, but they don’t”: exploring how new emergency medicine consultants experience uncertainty. Emerg Med J.

[19] Watsjold BK, Griffith M, Ilgen JS (2023). Stuck in the middle: the liminal experiences of entering practice. Emerg Med J.

[20] Parikh AB (2021). On the Transition to attendinghood. J Cancer Educ.

[21] Schrewe B (2018). Thrown into the world of independent practice: from unexpected uncertainty to new identities. Adv Health Sci Educ Theory Pract.

[22] Varpio L, Aschenbrener C, Bates J (2017). Tackling wicked problems: how theories of agency can provide new insights. Med Educ.

[23] Charmaz K, Charmaz K (2014). An invitation to Grounded Theory. Constructing Grounded Theory.

[24] Albert M, Mylopoulos M, Laberge S (2019). Examining grounded theory through the lens of rationalist epistemology. Adv Health Sci Educ Theory Pract.

[25] Mylopoulos M, Regehr G, Ginsburg S (2011). Exploring residents’ perceptions of expertise and expert development. Acad Med.

[26] Lajoie SP, Gube M (2018). Adaptive expertise in medical education: accelerating learning trajectories by fostering self-regulated learning. Med Teach.

[27] Panadero E (2017). A review of self-regulated learning: six models and four directions for research. Front Psychol Frontiers Media SA.

[28] Apramian T, Cristancho S, Watling C (2017). (Re)grounding grounded theory: a close reading of theory in four schools. Qualitative Research.

[29] Charmaz K (2006). Reconstructing theory in Grounded Theory studies. Constructing Grounded Theory, A Practical Guide Through Qualitative Analysis.

[30] Malterud K, Siersma VD, Guassora AD (2016). Sample size in qualitative interview studies. Qual Health Res.

[31] Westerman M, Teunissen PW, van der Vleuten CPM (2010). Understanding the transition from resident to attending physician: a transdisciplinary, qualitative study. Acad Med.

[32] Wiebe N, Hunt A, Taylor T (2024). “Everything new is happening all at once”: a qualitative study of early career obstetrician and gynaecologists’ preparedness for independent practice. Can Med Educ J.

[33] Cogbill TH, Shapiro SB (2016). Transition from training to surgical practice. Surg Clin North Am.

[34] De Leo AN, Drescher N, Bates JE (2022). Challenges in the transition to independent radiation oncology practice and targeted interventions for improvement. Tech Innov Patient Support Radiat Oncol.

[35] de Montbrun S, Patel P, Mobilio MH (2018). Am I cut out for this? Transitioning from surgical trainee to attending. J Surg Educ.

[36] Teunissen PW, Westerman M (2011). Opportunity or threat: the ambiguity of the consequences of transitions in medical education. Med Educ.

[37] Kilminster S, Zukas M, Quinton N (2011). Preparedness is not enough: Understanding transitions as critically intensive learning periods. Med Educ.

[38] Veysman BD (2010). Butchers move the meat; doctors care for patients. Ann Emerg Med.

[39] Auerbach L, Santen SA, Cutrer WB (2020). The educators’ experience: learning environments that support the master adaptive learner. Med Teach.

[40] Cutrer WB, Miller B, Pusic MV (2017). Fostering the development of master adaptive learners: a conceptual model to guide skill acquisition in medical education. Acad Med.

[41] Regan L, Hopson LR, Gisondi MA (2019). Learning to learn: a qualitative study to uncover strategies used by master adaptive learners in the planning of learning. Med Teach.

[42] Regan L, Hopson LR, Gisondi MA (2022). Creating a better learning environment: a qualitative study uncovering the experiences of master adaptive learners in residency. BMC Med Educ.

[43] Rich JV (2017). Proposing a Model of co-regulated learning for graduate medical education. Acad Med.

[44] Mylopoulos M, Steenhof N, Kaushal A (2018). Twelve tips for designing curricula that support the development of adaptive expertise. Med Teach.

[45] Ilgen JS, de Bruin ABH, Teunissen PW (2021). Supported Independence: the role of supervision to help trainees manage uncertainty. Acad Med.

[46] Dunbar-Yaffe R, Wu PE, Kay T (2022). Understanding the influence of the junior attending role on transition to practice: a qualitative study. J Grad Med Educ.

[47] Kaji A, Stevens C (2002). Moonlighting and the emergency medicine resident. Ann Emerg Med.

[48] Yama BA, Hodgins M, Boydell K (2018). A qualitative exploration: questioning multisource feedback in residency education. BMC Med Educ.

[49] Bolander Laksov K, Dornan T, Teunissen PW (2017). Making theory explicit - An analysis of how medical education research(ers) describe how they connect to theory. BMC Med Educ.

