# Supplementary material for: A Qualitative Study of Senior Residents’ Strategies to Prepare for Unsupervised Practice
Source: West J Emerg Med. 2025 Nov 26;26(6):1510–8. doi: 10.5811/westjem.48914 (PMC12698158; doi:10.5811/westjem.48914)
Supplement: Supplementary file 1 [file wjem-26-1510-s001.docx]

**Transition to Practice – Interview Guide**

**Permission to record?**

Thank you for taking time to speak with me today. My name is Max Griffith. I am a Medical Education Research Fellow at UW and I work clinically as an emergency medicine attending at UWMC and HMC.

I will be speaking with you today for a research project I am conducting. This study was granted exempt status by the IRB. Your participation is voluntary and there are no foreseeable risks. We’ll plan to talk for up to an hour, maybe less. If at any time you feel uncomfortable, we can skip a question or stop the interview. Everything said in this interview will be recorded, transcribed, and then de-identified so your views remain as confidential as possible.

I would like to understand your views of preparedness for practice after you graduate from residency, and how use your final months of clinical experience as a resident to prepare for this upcoming transition to practice (there are a few things you could call the transition from resident to attending; I am calling it the transition to practice).

Do you have any questions about the study or is there anything I can clarify?

1. To start, can you tell me about your career plans immediately after residency?
   - How are you feeling about this transition into practice? What emotions?
   - What challenges might you anticipate in this new practice setting? How does it compare to your current practice setting? How does it compare to previous practice settings?

~~2. I would like you to think of a challenge you recently had at work that you felt you would be ready to handle as an attending.~~

~~- What about this experience made you feel ready?~~

~~- How did you make that assessment?~~

~~3. Now think of a challenge at work recently that you felt less than ready to handle as an attending.~~

~~- What about this experience made you feel not ready?~~

~~- How did you make that assessment?~~

~~- What did you do in response to this experience?~~

1. Here is a question that might require a bit of time for reflection: Imagine your first shift as an attending. What is something you see that makes you feel unprepared?

- How does that imagined scenario make you feel?

- - What are you doing now to prepare for that kind of scenario?

1. What do you think it means for a graduating EM resident to be prepared for practice?

- Where did you get that picture from?

- - How do you determine whether you are approaching that mark? What feedback do you rely on?

1. Think about how you are approaching learning at work this year. Are you doing anything different knowing that you will soon be transitioning to an attending role?

~~- Has this been producing the results you hoped for?~~

~~- Have previous transitions influenced your approach?~~

~~- Are there any aspects of the resident work and training experience you especially~~

~~want to take advantage of before graduation?~~

- - How do you measure whether this has been working (in the moment and after the fact)?
  - What role do peers play? Attendings? Staff? Patients?
  - Tell me about a shift when you were micromanaged? When you could have used more support?
  - Are there any goals you have for your training before graduation? What are some things that support or get in the way of those goals?

1. How does this transition compare to past transitions in training?

1. Do you have any other thoughts on this topic that we did not already cover?

Text in normal font indicates content from the initial interview guide that remained unchanged

Highlighted text indicates content that was iteratively added to the interview guide

~~Strikethrough~~ text indicates content that was iteratively removed from the interview guide
